# Human Activity, not Ecosystem Characters, Drives Potential Species Invasions

**DOI:** 10.1371/journal.pbio.0060039

**Published:** 2008-02-05

**Authors:** Richard Robinson

From the Asian tiger mosquito in the American South, to the Eurasian zebra mussel in the Great Lakes, to European quackgrass throughout the United States, invasions of non-native species can disrupt ecosystems, cause havoc with local economies, and even threaten health. A new study shows that, at least for freshwater fishes, the major driver of successful invasion is human development, not intrinsic ecological factors, suggesting that in the future, many more newcomers will be making their homes in foreign lands.

Competing hypotheses have been proposed to account for the establishment of non-native species. Human activities, from disrupting ecosystems to transporting exotic species, have clearly contributed to many invasions. But do ecosystems themselves play a part? The “biotic resistance” hypothesis suggests that species-rich environments can deter newcomers, while the “biotic acceptance” hypothesis suggests the opposite, that if it's good for the locals, it's good for the invaders.

To test the relative weight of human versus other ecologic factors on freshwater fish invasions, Fabien Leprieur and colleagues collated species data on over 1,000 river basins throughout the world (collectively draining over 80% of Earth's land surface), characterizing each species as native or non-native.

**Figure:**
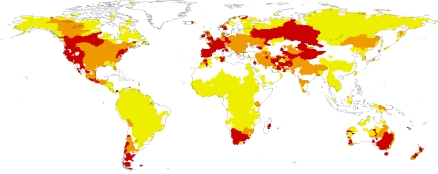
Global hot spots of freshwater fish invasion.

They found six “hot spots” of invasion: the Pacific coast of North and Central America, southern South America, western and southern Europe, central Eurasia, South Africa and Madagascar, southern Australia, and New Zealand. In these areas, more than a quarter of species were non-native, and they had higher-than-average numbers of native species threatened by extinction.

There was no correlation between the richness of native species and that of non-natives, at once refuting biotic acceptance (for which high numbers of natives predict high numbers of non-natives) and biotic resistance (for which high numbers of natives predict low numbers of non-natives). Instead, regardless of local diversity, non-natives were most abundant where human activity was greatest: gross domestic product, population density, and percentage of urban area predicted the greatest proportion of invasives throughout the globe.

The galloping pace of economic development throughout the world during the 20th century, and the likely increase of that pace in the developing world during the 21st century, suggest that the invasive species problem will get worse, not better. To mitigate this problem, the authors suggest that their data be used to anticipate where new invasions are likely and that priority be given to heading off new invaders before they are established in those areas. They also recommend a ban on the import of non-natives into invasion hot spots without detailed impact assessments.

